# Supplementary material for: Identification of critical genetic variants associated with metabolic phenotypes of the Japanese population
Source: Commun Biol. 2020 Nov 11;3:662. doi: 10.1038/s42003-020-01383-5 (PMC7659008; doi:10.1038/s42003-020-01383-5)
Supplement: Supplementary file 1 — Supplementary Information [file 42003_2020_1383_MOESM1_ESM.pdf]

Supplementary Information

Seizo Koshiba, Ikuko N. Motoike, Daisuke Saigusa, Jin Inoue, Yuichi Aoki, Shu Tadaka, Matsuyuki Shirota, Fumiki Katsuoka, Gen Tamiya, Naoko Minegishi, Nobuo Fuse, Kengo Kinoshita, Masayuki Yamamoto

Identification of critical genetic variants associated with metabolic phenotypes of the Japanese population

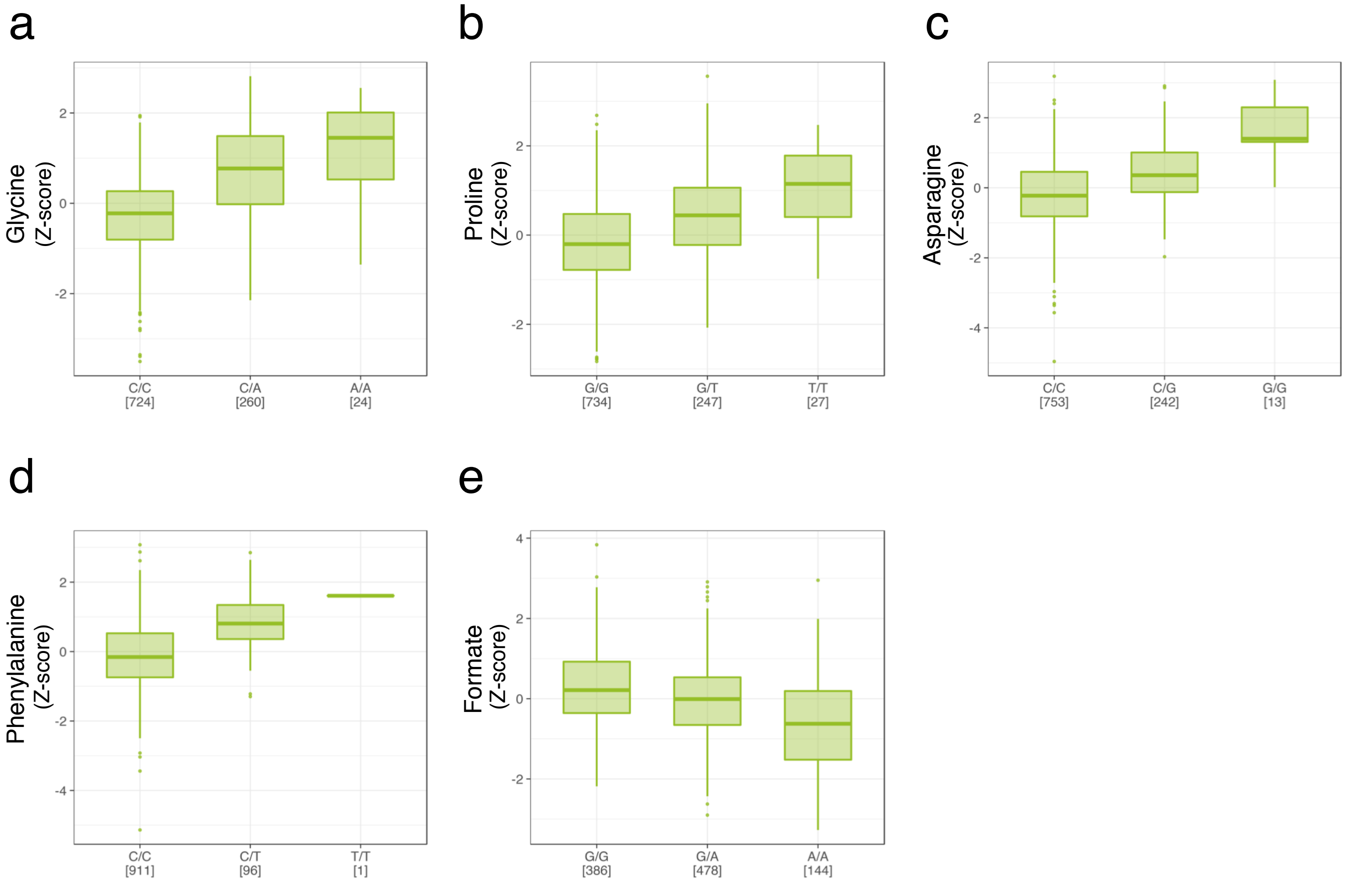

Supplementary Fig. 1: Distribution of the Plasma Metabolites.
Distributions of the plasma metabolites across the genotypes were shown using a box plot. These figures were made using R package.

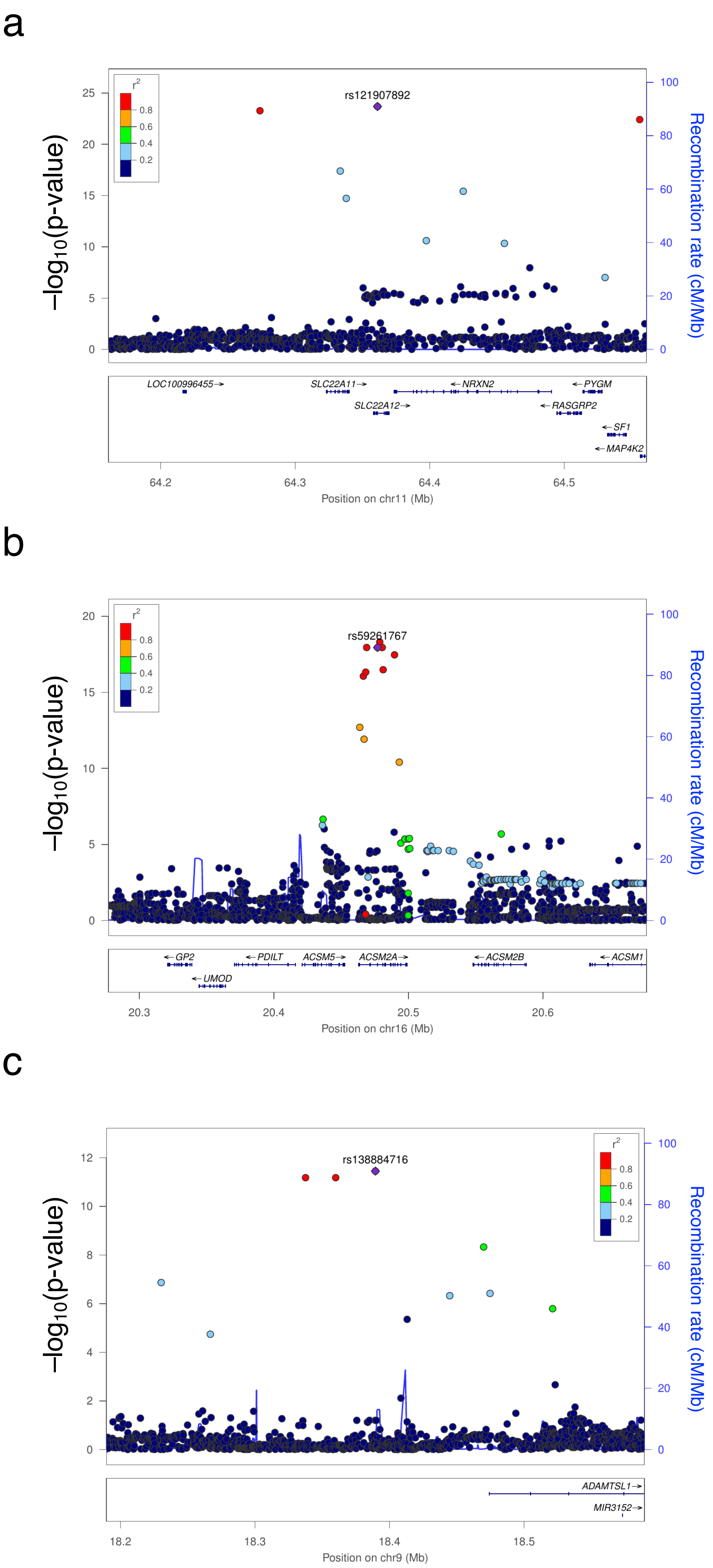

Supplementary Fig. 2

d

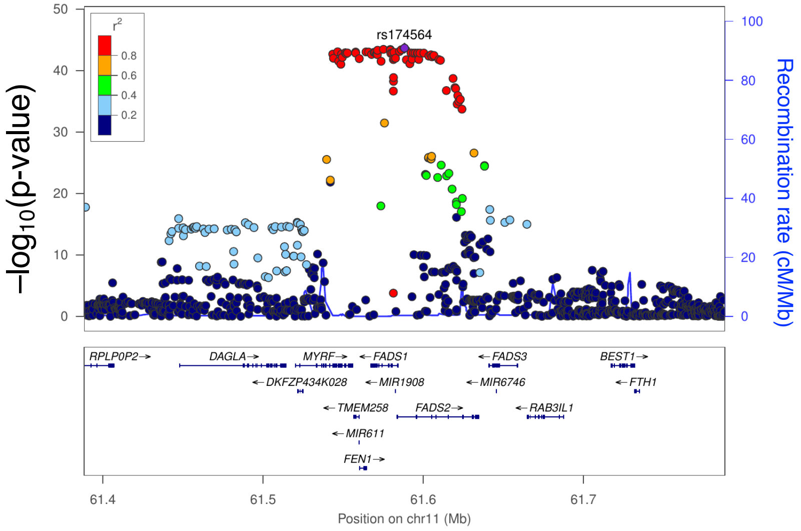

rs174564
r2
0.8
0.6
0.4
0.2
−log10(p-value)
Recombination rate (cM/Mb)
RPLP0P2
DAGLA
MYRF
FADS1
FADS3
BEST1
DKFZP434K028
MIR1908
MIR6746
FTH1
TMEM258
FADS2
RAB3IL1
MIR611
FEN1
Position on chr11 (Mb)

e

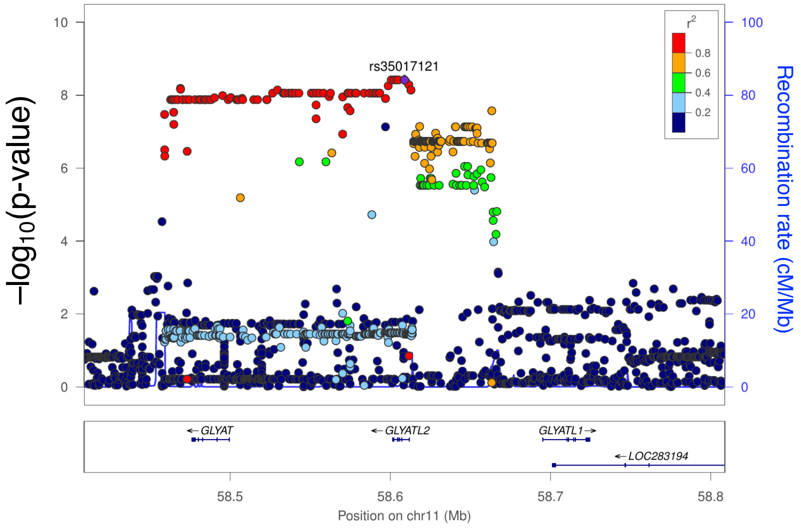

rs35017121
r2
0.8
0.6
0.4
0.2
−log10(p-value)
Recombination rate (cM/Mb)
GLYAT
GLYATL2
GLYATL1
LOC283194
Position on chr11 (Mb)

f

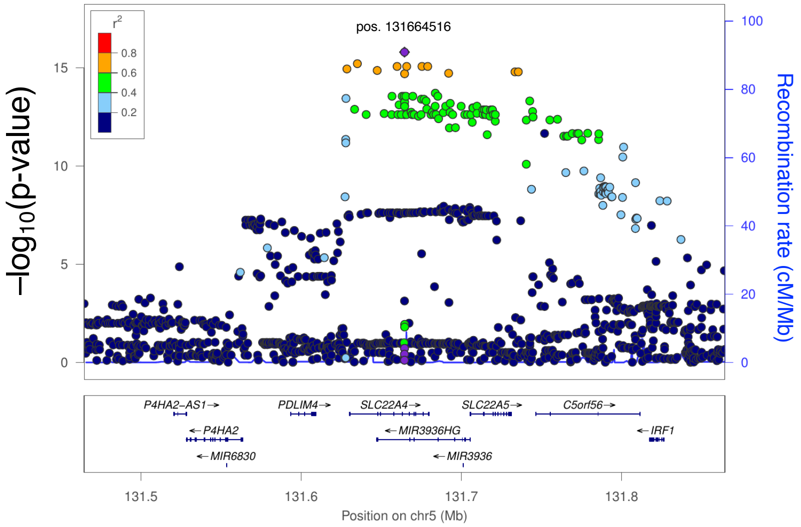

pos. 131664516
r2
0.8
0.6
0.4
0.2
−log10(p-value)
Recombination rate (cM/Mb)
P4HA2-AS1
PDLIM4
SLC22A4
SLC22A5
C5orf56
P4HA2
MIR3936HG
IRF1
MIR6830
MIR3936
Position on chr5 (Mb)

g

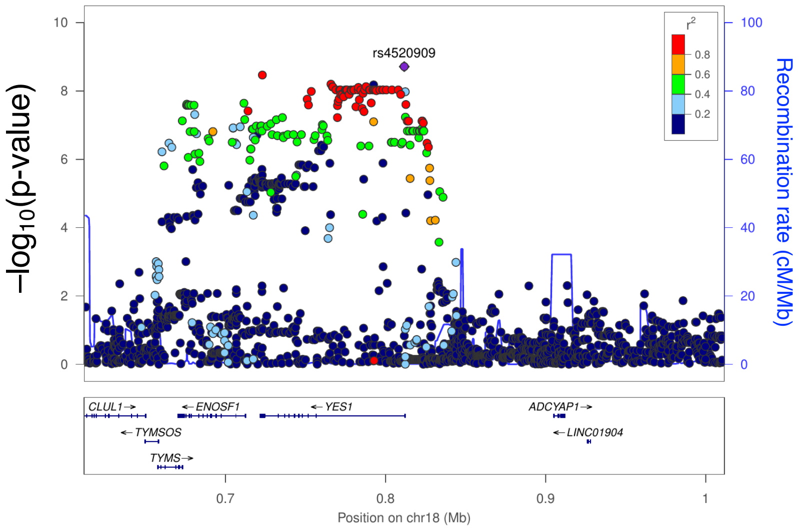

h

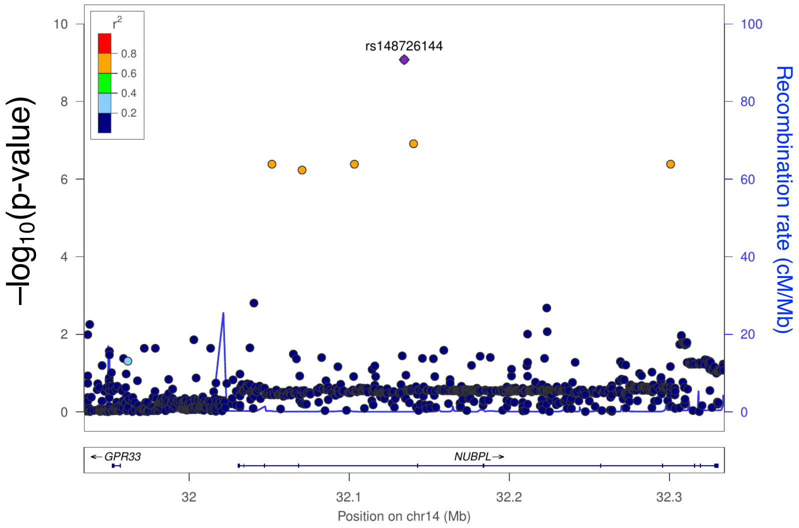

Supplementary Fig. 2

Supplementary Fig. 2: Regional association plots for the loci.
Statistical significance of associated SNPs are plotted on the $-\log_{10}$(P-value) scale as a function of chromosomal position (NCBI 37). The identified causal SNP at each locus is shown in purple. Correlation of the causal SNP to other SNPs at each locus is shown on a scale from minimal (blue) to maximal (red).

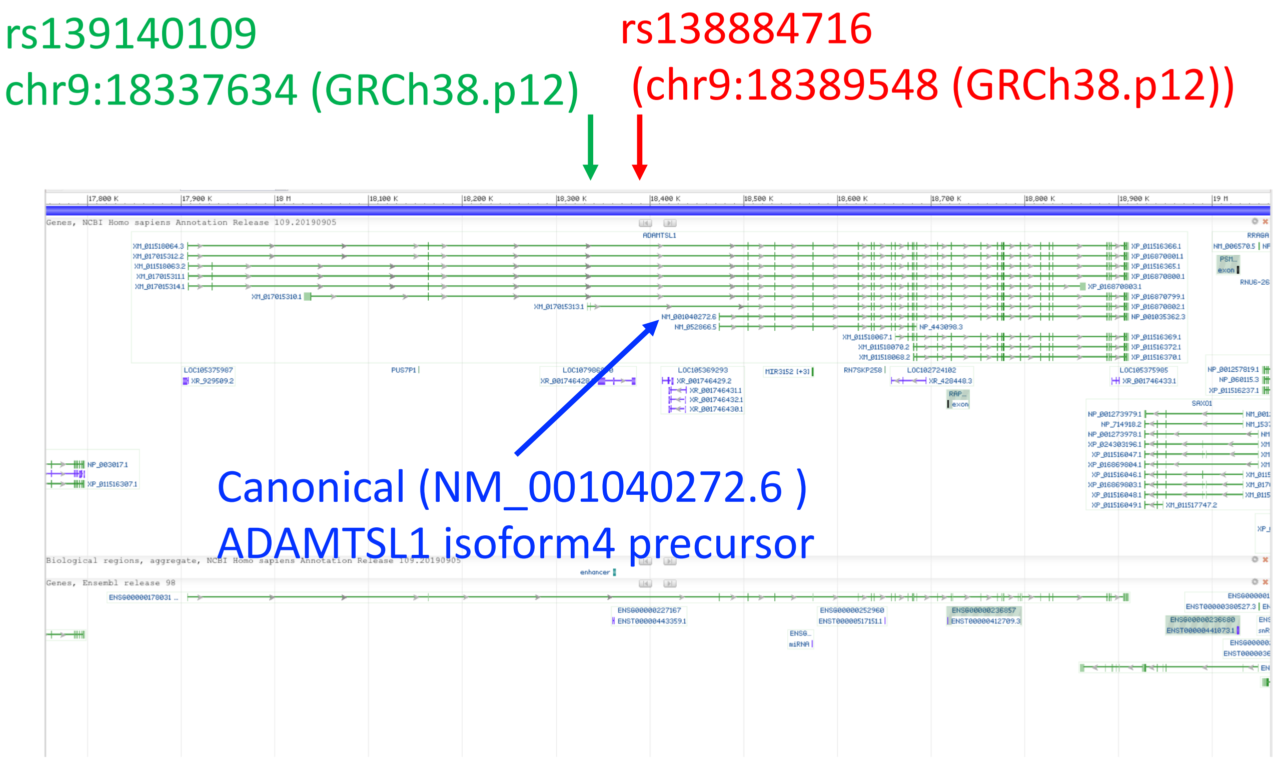

https://www.ncbi.nlm.nih.gov/gene/92949

Supplementary Fig. 3: Genomic region of *ADAMTSL1* gene (GRCh38), quoted from the description of *ADAMTSL1* gene (Gene ID 92949) in the National Center for Biotechnology Information (NCBI) database. A number of transcripts and translated proteins are annotated. The SNP positions identified in this MGWAS study were shown in red and green arrows. The canonical product from *ADAMTSL1* gene was shown in a blue arrow.

a

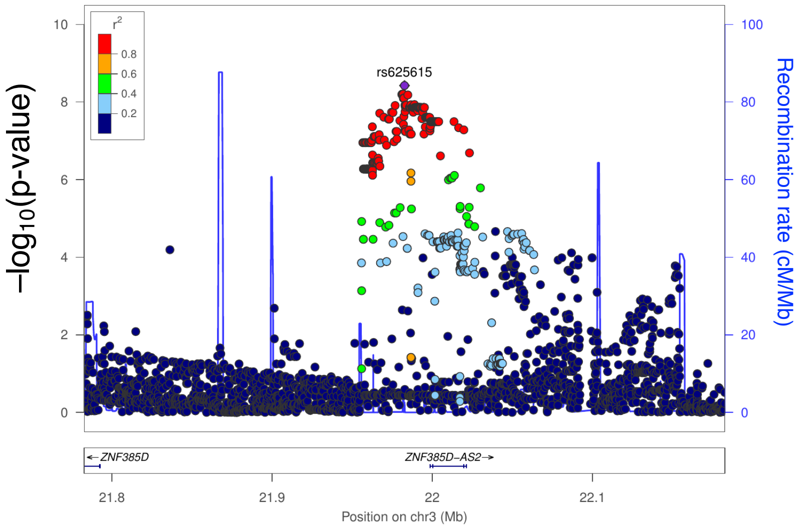

b

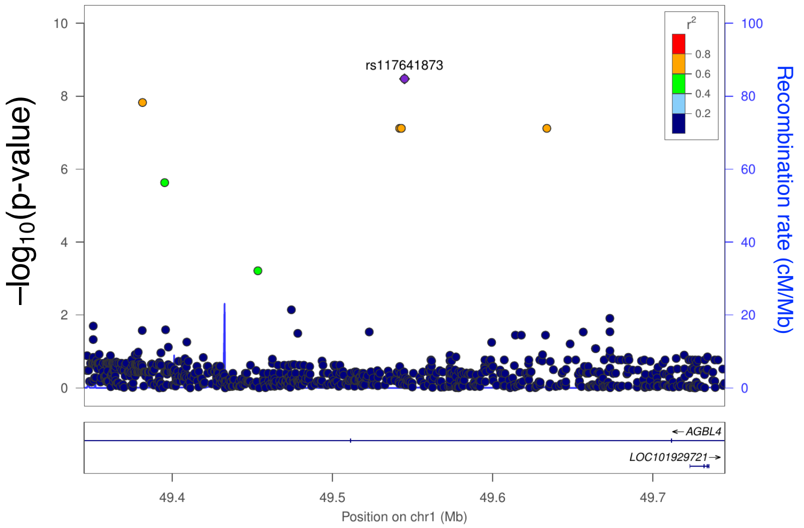

Supplementary Fig. 4: Regional association plots for the loci in female population. Significant associations identified in female population were represented.

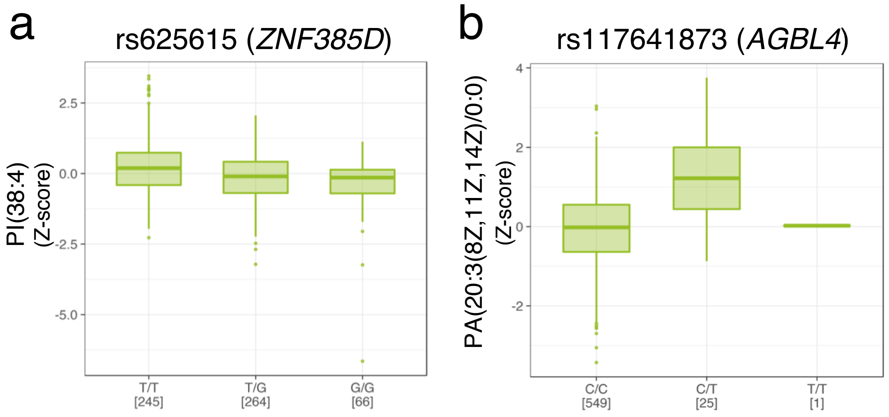

Supplementary Fig. 5: Distribution of the plasma metabolites in female population. Significant associations identified in female population were represented.

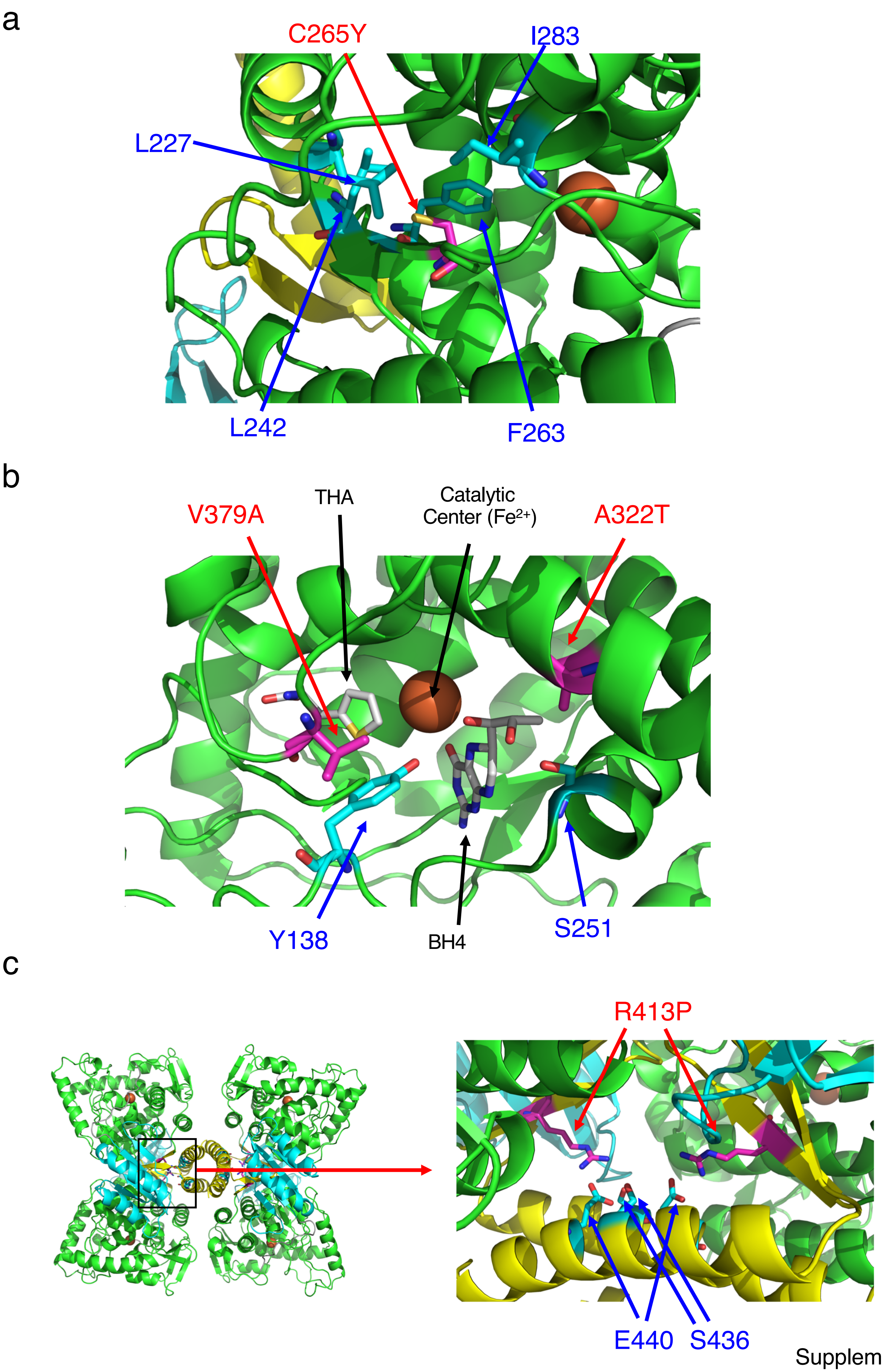

Supplementary Fig. 6

Supplementary Fig. 6: Structure of PAH.

Structural details around the residues (a) C265, (b) V379 & A322, and (c) R413 are shown. The catalytic, regulatory, and tetramerization domains are depicted in green, cyan, and yellow. The residues around C265, V379, A322, and R413 are shown by stick models. The residues C265, V379, A322, and R413 are labeled in red, while others are labeled by cyan. (a) The side chain of C265 locates in the hydrophobic core of PAH, indicating the substitution of C265 to tyrosine results to the disruption of the core. (b) V379 and A322 locate in the substrate binding regions near the catalytic center. Hence, the coordinate of the substrate-bound form of PAH (PDB ID: 1MMK) was used in this figure, because the substrate binding causes the structural change around the catalytic center. In the complex form, the residue Y138 moves from a surface position to a buried position at the active site and interacts with V379, indicating the substitution of V379 to alanine disrupt this interaction, resulting to destabilize the structure of active form. On the other hand, the residue A322 locates near cofactor BH4 and the residue S251, indicating that the substitute of A322 to threonine perturbs them. (c) R413 locates in the tetramerization domain and the sidechain of R413 interacts with those of S436 and E440, indicating that the substitution of R413 to proline disrupts these interactions, resulting to destabilize tetramerization of PAH.

Supplementary
Fig. 6

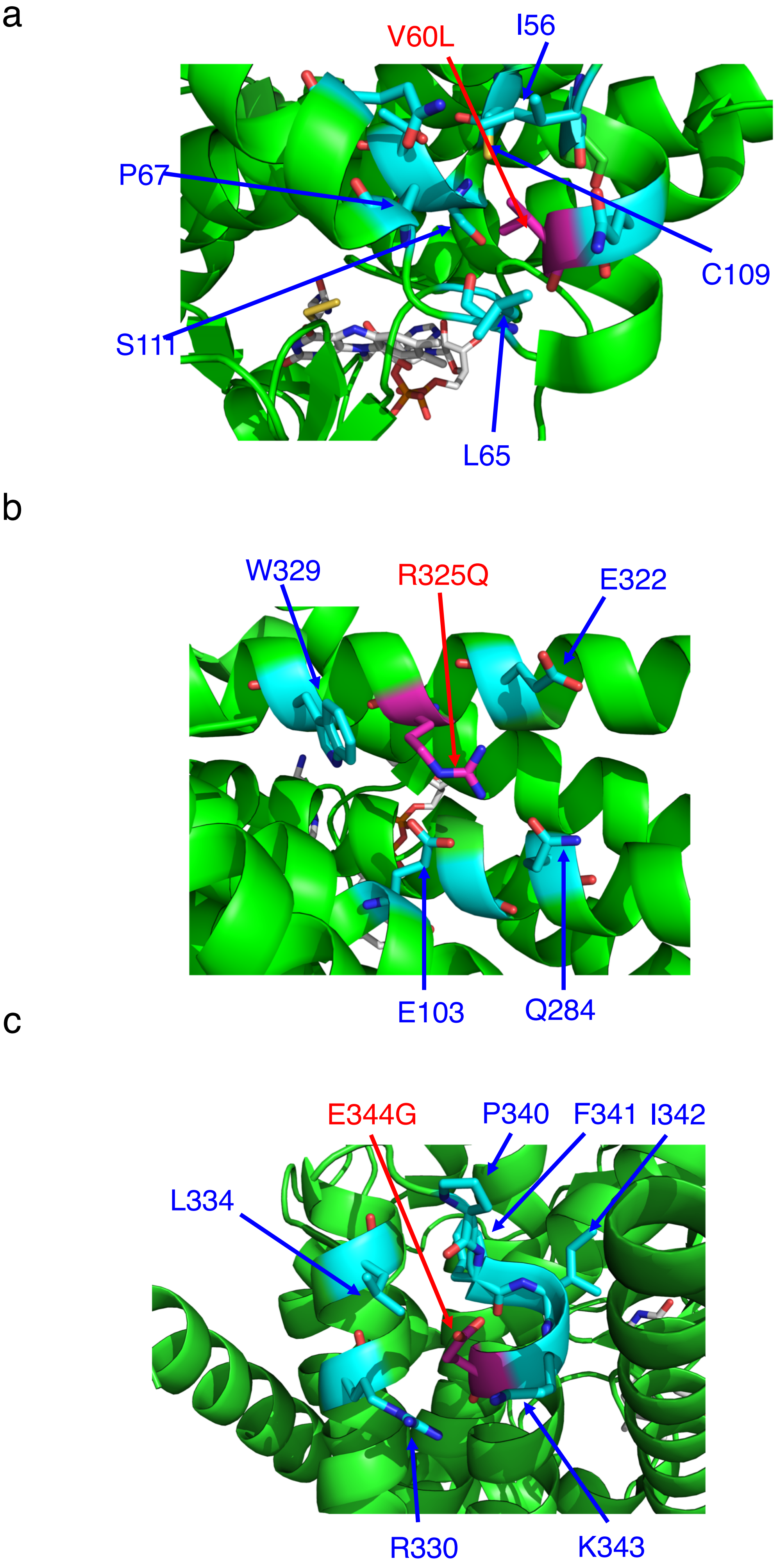

Supplementary Fig. 7

Supplementary Fig. 7: Structure of ACADS.
Structural details around the residues (a) V60, (b) R325, and (c) E344 are shown. The residues around V60, R325, and E344 are shown by stick models. The residues V60, R325, and E344 are labeled in red, while others are labeled by cyan.

Supplementary
Fig. 7

a

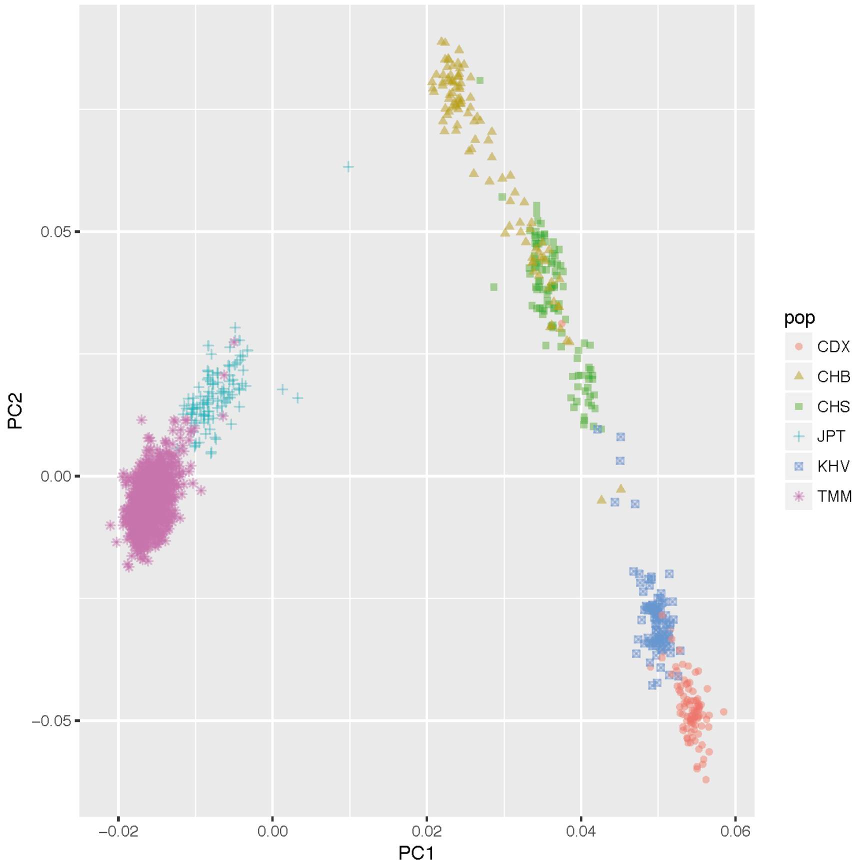

b

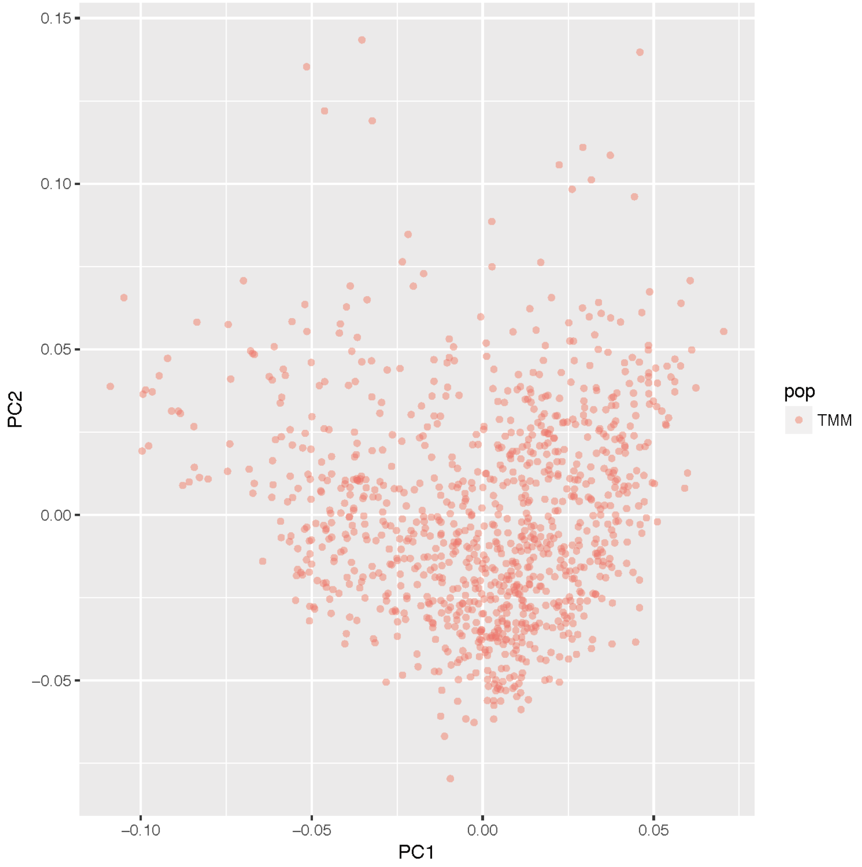

Supplementary Fig. 8

Supplementary Fig. 8: Population structure of 1,008 individuals in this MGWAS.
(a) The score plot of 1,008 individuals in this study (depicted TMM) with East Asian populations in the 1000 Genomes Project (Phase3) by PCA. The East Asian populations are as follows: CHB (Han Chinese in Beijing, China), JPT (Japanese in Tokyo, Japan), CHS (Southern Han Chinese), CDX (Chinese Dai in Xishuangbanna, China), KHV (Kinh in Ho Chi Minh City, Vietnam).
(b) The score plot of only 1,008 ToMMo individuals by PCA.

Data processing of PCA: The genotype dataset of 1,008 individuals was constructed by eliminating variants on the basis of the following criteria: MAF < 0.01 or HWE < 1.0e-5. The genotype dataset of East Asian populations was constructed from the 1000 Genomes Phase3 genotype data in the same manner. After integrating these datasets by selecting common variants, the combined dataset was refined by eliminating variants with MAF < 0.05 or HWE < 0.05 or those with a missing rate > 0.01. This dataset was applied for PCA after LD pruning for the remaining variants with PLINK by selecting the "--indep-pairwise 1500 1000 0.03" option.

a

C18pos

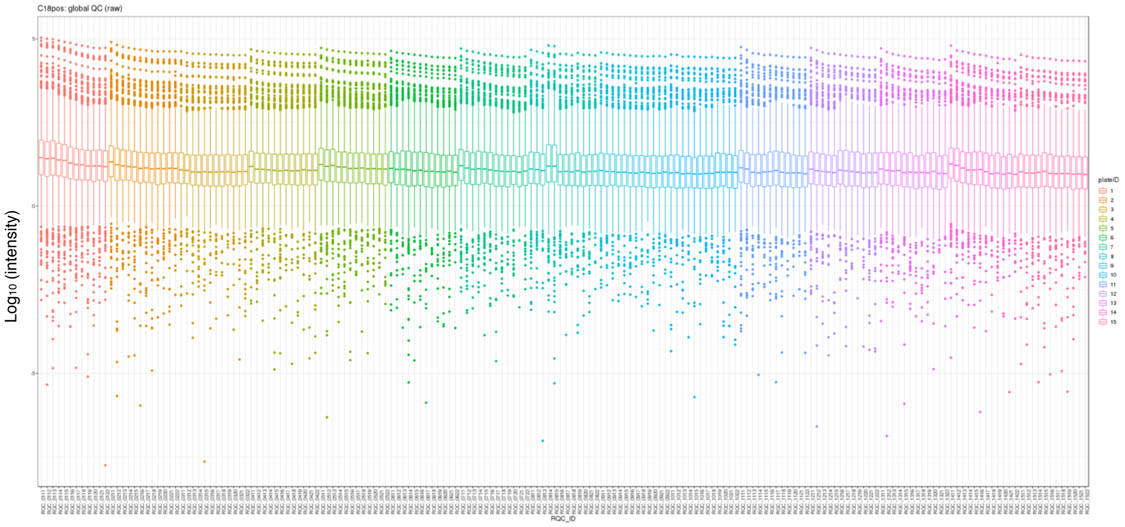

HILICneg

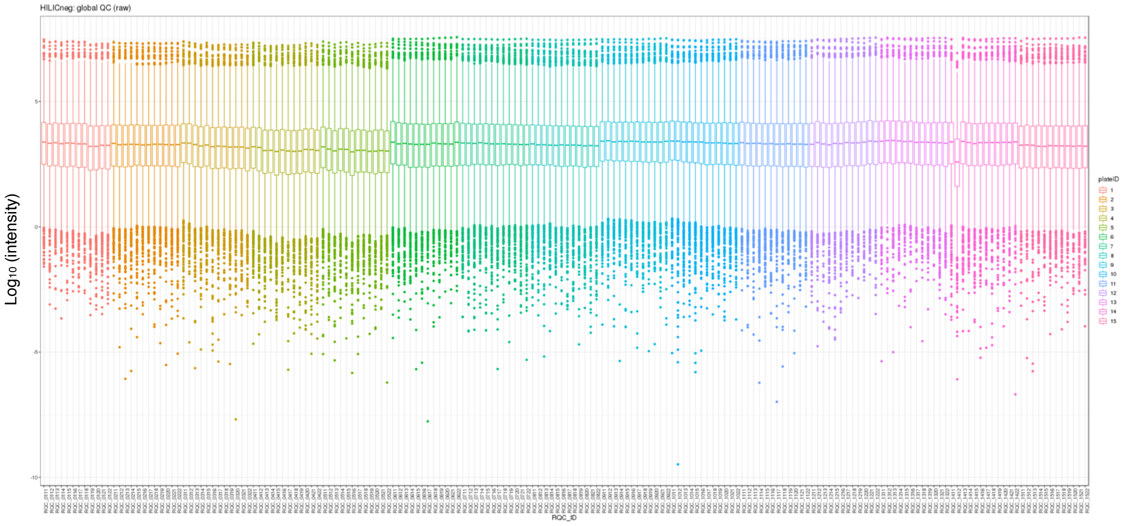

b

C18pos

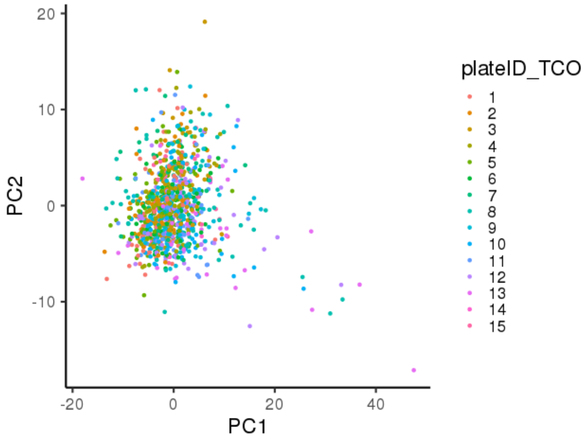

HILICneg

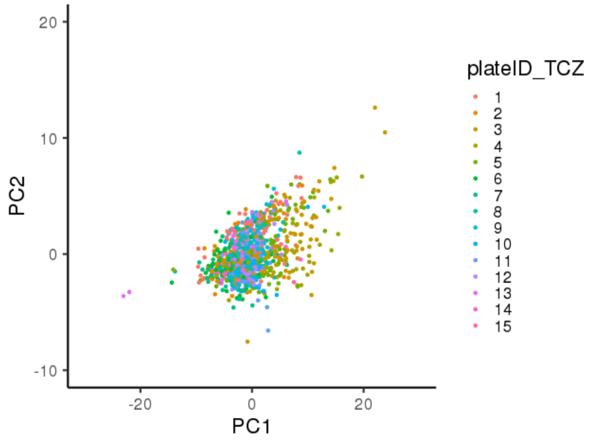

Supplementary Fig. 9: Box plots of gQC before normalization and score plots of PCA after normalization: C18pos. and HILICneg. modes of MS analysis.
(a) Box plots of 180 reference (global) quality control (gQC) plasma samples among 15 plates of the 1,008 individual plasma analyses at the C18pos and HILICneg modes. The log values of intensity are shown as follows; boxes represent the interquartile range (IQR) between the first quantile (Q1) and third quartiles (Q3), and the line inside represents the median. Whiskers denote the lowest and highest values within 1.5 × IQR from Q1 and Q3, respectively. Colored dots represent outliers beyond the whiskers.
(b) Score plots of 1,008 individuals among 15 plates by PCA at the C18pos and HILICneg modes. The values of intensity after normalization based on the gQC values were used for the PCA using the R package (ver. 3.4.2) with the scaling option. (Left panel) Dots represent the individuals at the C18 pos mode. (Right panel) Dots represent the individuals at the HILICneg mode (For this plot, we excluded one outlier affected by an artifact from the MS analysis, and thus a total 1,007 individuals were plotted for the HILICneg mode). For both panels, 15 colors are shown as the plate difference.

Supplementary Notes

Details of the associations between metabolites and SNPs observed in only the female population

One of the phosphatidyl inositol (PI) family members, PI(38:4), was closely associated with genetic variants in the ZNF385D gene, and we found that this association was significant only in female (Table 2 and Supplementary Fig 4a, 5a). The ZNF385D gene encodes a zinc finger protein expressed in a wide variety of tissues, such as the testis and brain. While the molecular function of the ZNF385D protein remains unknown, this protein was shown to be involved in various neurocognitive disorders, such as schizophrenia, reading disability, and language impairment[1,2]. PI family members are abundantly expressed in brain tissue and involved in cell signaling and membrane trafficking, suggesting that genetic variations of ZNF385D may affect PI signaling pathways in brain tissue. In addition, previous studies reported that the ZNF385D locus is associated with many kinds of phenotypes, such as cardiovascular diseases, cholesterol, phospholipids, and pulmonary diseases, suggesting that ZNF385D variants influence many kinds of functions (Supplementary Data 4).

We also identified an association of the phospholipid PA(20:3(8Z,11Z,14Z)/0:0) with the genetic variants located in the ATP/GTP binding protein-like 4 gene (AGBL4) specifically in the female population (Table 2 and Supplementary Figs 4b and 5b). AGBL4, also known as CCP6, mediates the deglutamylation of proteins[3]. To the best of our knowledge, this is the first report to show that a genetic variant of this gene is directly associated with increased levels of phospholipid molecules. In this regard, relationships of this gene and diseases have been documented: genetic variants of AGBL4 are associated with anti-tuberculosis drug-induced liver toxicity[4], and hypermethylation of the promoter region of the gene is associated with colorectal cancer[5]. Genetic variants of this gene were also shown to be associated with the concentrations of serum lipids, such as total cholesterol, triglyceride, and high-density lipoprotein (HDL) cholesterol (Supplementary Data 4)[6,7].

Supplementary References

1. Xu, C. et al. BCL9 and C9orf5 are associated with negative symptoms in schizophrenia: meta-analysis of two genome-wide association studies. PLoS One 8, e51674 (2013).
2. Eicher, J.D. et al. Genome-wide association study of shared components of reading disability and language impairment. Genes Brain Behav 12, 792-801 (2013).
3. Rodriguez de la Vega Otazo, M., Lorenzo, J., Tort, O., Aviles, F.X. & Bautista, J.M. Functional segregation and emerging role of cilia-related cytosolic carboxypeptidases (CCPs). FASEB J 27, 424-31 (2013).
4. Petros, Z. et al. Genome-wide association and replication study of anti-tuberculosis drugs-induced liver toxicity. BMC Genomics 17, 755 (2016).
5. Lin, P.C. et al. Clinical Relevance of Plasma DNA Methylation in Colorectal Cancer Patients Identified by Using a Genome-Wide High-Resolution Array. Ann Surg Oncol 22 Suppl 3, S1419-27 (2015).
6. Guo, T. et al. Integrative mutation, haplotype and G x G interaction evidence connects ABGL4, LRP8 and PCSK9 genes to cardiometabolic risk. Sci Rep 6, 37375 (2016).
7. Guo, T. et al. AGBL4, PRL8 and PCSK9 genetic variants and their interactions on dyslipidemia. Int. J. Clin. Exp. Pathol. 10, 2652-2674 (2017).
