# Supplementary material for: Identification of critical genetic variants associated with metabolic phenotypes of the Japanese population
Source: Commun Biol. 2020 Nov 11;3:662. doi: 10.1038/s42003-020-01383-5 (PMC7659008; doi:10.1038/s42003-020-01383-5)
Supplement: Supplementary file 3 — Description of Additional Supplementary Files [file 42003_2020_1383_MOESM3_ESM.docx]

**Description of Supplementary Data**

**Supplementary Data 1**. Summary for the metabolites used for this MGWAS study.

**Supplementary Data 2**. Comparison of the associations identified in this MGWAS analysis with those identified in other MGWAS analyses.

**Supplementary Data 3**. Comparison of allele frequencies of the genetic variants identified in this MGWAS study for the Japanese population with those in other populations.

**Supplementary Data 4**. Information of the results of previous association studies (PheGenI) or disease reports (OMIM, PubMed) for the associated genes identified in this MGWAS study.

**Supplementary Data 5**. Source data underlying boxplots shown in the Main Figures (Figs 1b-1i, 2b, 2d, 2f, 2h, 2j, 2l, 2n, 4a, 4c).
