# Supplementary material for: Identification of critical genetic variants associated with metabolic phenotypes of the Japanese population
Source: Commun Biol. 2020 Nov 11;3:662. doi: 10.1038/s42003-020-01383-5 (PMC7659008; doi:10.1038/s42003-020-01383-5)
Supplement: Supplementary file 9 — Reporting Summary [file 42003_2020_1383_MOESM9_ESM.pdf]

nature research

Corresponding author(s): Masayuki Yamamoto

Last updated by author(s): Sep 11, 2020

Reporting Summary

Nature Research wishes to improve the reproducibility of the work that we publish. This form provides structure for consistency and transparency in reporting. For further information on Nature Research policies, see our Editorial Policies and the Editorial Policy Checklist.

Statistics

For all statistical analyses, confirm that the following items are present in the figure legend, table legend, main text, or Methods section.

n/a	Confirmed	
☐	☒	The exact sample size (*n*) for each experimental group/condition, given as a discrete number and unit of measurement
☐	☒	A statement on whether measurements were taken from distinct samples or whether the same sample was measured repeatedly
☐	☒	The statistical test(s) used AND whether they are one- or two-sided<br>*Only common tests should be described solely by name; describe more complex techniques in the Methods section.*
☐	☒	A description of all covariates tested
☐	☒	A description of any assumptions or corrections, such as tests of normality and adjustment for multiple comparisons
☐	☒	A full description of the statistical parameters including central tendency (e.g. means) or other basic estimates (e.g. regression coefficient) AND variation (e.g. standard deviation) or associated estimates of uncertainty (e.g. confidence intervals)
☐	☒	For null hypothesis testing, the test statistic (e.g. *F*, *t*, *r*) with confidence intervals, effect sizes, degrees of freedom and *P* value noted<br>*Give P values as exact values whenever suitable.*
☒	☐	For Bayesian analysis, information on the choice of priors and Markov chain Monte Carlo settings
☒	☐	For hierarchical and complex designs, identification of the appropriate level for tests and full reporting of outcomes
☒	☐	Estimates of effect sizes (e.g. Cohen's *d*, Pearson's *r*), indicating how they were calculated

Our web collection on statistics for biologists contains articles on many of the points above.

Software and code

Policy information about availability of computer code

Data collection	Metabolites data was obtained using TopSpin 2.1 (Bruker), Chenomx NMR Suite 8.0 (Chenomx), Progenesis QI v2.3 data analysis software (Nonlinear Dynamics).
Data analysis	GWAS analysis was performed by PLINK1.9b5.1. Metabolite and covariate data transformation was performed by R package (car ver.2.1.5). Annotation of genes was performed by ANNOVAR (ver. 2017Jul16).

For manuscripts utilizing custom algorithms or software that are central to the research but not yet described in published literature, software must be made available to editors and reviewers. We strongly encourage code deposition in a community repository (e.g. GitHub). See the Nature Research guidelines for submitting code & software for further information.

Data

Policy information about availability of data

All manuscripts must include a data availability statement. This statement should provide the following information, where applicable:

- Accession codes, unique identifiers, or web links for publicly available datasets
- A list of figures that have associated raw data
- A description of any restrictions on data availability

Summary GWAS statistics are publicly available at Japanese Multi Omics Reference Panel website (https://jmorp.megabank.tohoku.ac.jp/). Individual's genotyping results and metabolite data used for the association study are available upon request after approval of the Ethical Committee and the Materials and Information Distribution Review Committee of Tohoku Medical Megabank Organization.

April 2020

Field-specific reporting

Please select the one below that is the best fit for your research. If you are not sure, read the appropriate sections before making your selection.

☒ Life sciences ☐ Behavioural & social sciences ☐ Ecological, evolutionary & environmental sciences

For a reference copy of the document with all sections, see nature.com/documents/nr-reporting-summary-flat.pdf

Life sciences study design

All studies must disclose on these points even when the disclosure is negative.

Sample size	We selected 1,008 adult participants from 3,552 participants whose whole-genome sequences had already been obtained.
Data exclusions	We filtered out close relatives of cohort participants from samples for analysis.
Replication	For a replication study, we selected additional 295 participants (130 female) and performed MGWAS using whole-genome sequence data and metabolome data in a similar manner to the discovery study.
Randomization	We randomly selected 1,008 participants from 3,552 participants (3.5KJPNv2). The ratio of sex was nearly equal with that of our cohort studies.
Blinding	Samples and informations obtained from cohort participants were anonymized before analysis.

Reporting for specific materials, systems and methods

We require information from authors about some types of materials, experimental systems and methods used in many studies. Here, indicate whether each material, system or method listed is relevant to your study. If you are not sure if a list item applies to your research, read the appropriate section before selecting a response.

Materials & experimental systems

n/a	Involved in the study	
☒	☐	Antibodies
☒	☐	Eukaryotic cell lines
☒	☐	Palaeontology and archaeology
☒	☐	Animals and other organisms
☐	☒	Human research participants
☒	☐	Clinical data
☒	☐	Dual use research of concern

Methods

n/a	Involved in the study	
☒	☐	ChIP-seq
☒	☐	Flow cytometry
☒	☐	MRI-based neuroimaging

Human research participants

Policy information about studies involving human research participants

Population characteristics	Our cohort project conducts population-based prospective cohort studies in Japan.
Recruitment	We recruit participants on the sites for specific health checkups of the annual community health examination. Additionally, we have established facilities of seven "Community Support Centers" in Miyagi Prefecture for the voluntary admission-type recruitment and health assessment of participants.
Ethics oversight	The ethics committee of Tohoku University

Note that full information on the approval of the study protocol must also be provided in the manuscript.
